# Supplementary material for: Clinical Characteristics and Management of Patients with a Suspected COVID-19 Infection in Emergency Departments: A European Retrospective Multicenter Study
Source: J Pers Med. 2022 Dec 19;12(12):2085. doi: 10.3390/jpm12122085 (PMC9787691; doi:10.3390/jpm12122085)
Supplement: Supplementary file 1 [file jpm-12-02085-s001.zip › jpm-1971994-supplementary.pdf]

† **EUSEM Research network study group:** (EUROCOV Investigators list):

[12] **C**Visnja Nesek Adam, Resuscitation and Intensive Care, University Department of Anesthesiology, Sveti Duh, University Hospital, 10000 Zagreb, Croatia

[13] Ivan Jurić, Resuscitation and Intensive Care, University Department of Anesthesiology, Sveti Duh, University Hospital, 10000 Zagreb, Croatia

[14] Jari Nyrhilä, Emergency Department, Division of Medicine, Seinäjoki Central Hospital, 60220 Seinäjoki, Finland

[15] Anu Pakkanen, Emergency Department, Division of Medicine, Kanta-Häme Central Hospital, 13530 Hämeenlinna, Finland

[16] Justine Clément, School of Medicine, Tours University, 37000 Tours, France

[17] Mustapha Sebbane , Emergency Department, Lapeyronie University Hospital, 34000 Montpellier, France

[18] Damien Viglino, emergency Department, Grenoble-Alpes University Hospital, 38700 La Tronche, France

[19] Nicolas Marjanovic, Emergency department, Poitiers University Hospital, 86000 Poitiers, France

[20] Richard Macrez, Emergency Department, Caen Normandie University Hospital, 14000 Caen, France

[21] Tania Marx, Department of Emergency Medicine & Critical Care University of Franche-Comté, Medical & Trauma Center, 25000 Besançon, France

[22] Abdo Khoury, Department of Emergency Medicine & Critical Care University of Franche-Comté, Medical & Trauma Center, 25000 Besançon, France

[23] Farés Moustafa, Emergency Department, University Hospital, 63000 Clermont-Ferrand, France

[24] Tahar Chouihed, Emergency Department, Lorraine University Hospital, 54000 Nancy, France

[25] Cédric Gil-Jardine, Emergency Department, Pellegrin University Hospital, 33000 Bordeaux, France

[26] Martin Möckel, Departments of Emergency and Acute Medicine, Campus Mitte, Virchow-Klinikum Charité-Universitätsmedizin, 10117 Berlin, Germany

[27] Ifantis Dimitrios, Emergency Medicine Department, Attikon University Hospital, Athens, 12462, Greece

[28] Mata Tsikrika, Emergency Medicine Department, Attikon University Hospital, Athens, 12462, Greece

[28] Filippo Manelli, Emergency Department, Brescia University Hospital, 25123 Brescia, Italia

[29] Francesco Rocco Pugliese, Emergency Department, Roma University Hospital, 00159 Roma, Italia

Steering Committee:

Said Laribi (Chair: France, CHU Tours), Anthony Chauvin (Lariboisiere Hospital, APHP, Paris, France) Visnja Nesek Adam (Croatia, Clinical hospital Sveti Duh), Ari Palomaki (Finland, Kanta-Häme Central Hospital and Tampere University), Effie Polyzogopoulou (Greece, University General Hospital Attikon), Andrea Fabbri (Italy, AUSL Romagna), Lars Peter Bjornsen (Norway), Anna Slagman (Germany).

Local investigators:

Croatia: Visnja Nesek Adam (Clinical hospital Sveti Duh, Zagred), Ivan Jurić (Clinical hospital Sveti Duh, Zagred)

Finland: Ari Palomäki (Kanta-Häme Central Hospital, Hämeenlinna), Jari Nyrhilä (Seinäjoki Central Hospital, Seinäjoki), Anu Pakkanen (Kanta-Häme Central Hospital, Hämeenlinna).

France: Said Laribi and Justine Clément (CHU Tours), Mustapha Sebbane (CHU Montpellier), Damien Viglino (CHU Grenoble), Nicolas Marjanovic (CHU de Poitiers), Richard Macrez (CHU de Caen), Tania Marx and Abdo Khoury (CHU de Besançon), Farés Moustafa (CHU de Clermont Ferrand), Tahar Chouihed (CHU de Nancy), Cédric Gil-Jardine (CHU de Bordeaux).

Germany: Martin Möckel (Charite Universitätsmedizin Berlin, Berlin).

Greece: Effie Polyzogopoulou (University General Hospital Attikon, Athens), Ifantis Dimitrios (Sotiria General Hospital, Athens).

Italy: Andrea Fabbri (AUSL Romagna, Forli), Filippo Manelli (ASST Valcamonica, Brescia), Francesco Rocco Pugliese (Dipartimento Emergenza Urgenza ASL Roma 2, Roma).

Norway: Lars Peter Bjornsen (St. Olav's Hospital -Trondheim University hospital, Trondheim).
